# Cost Analysis of Magnetic Resonance Imaging and Computed Tomography in Cardiology: A Case Study of a University Hospital Complex in the Euro Region

**DOI:** 10.3390/healthcare11142084

**Published:** 2023-07-21

**Authors:** Francisco Reyes-Santias, Carlos García-García, Beatriz Aibar-Guzmán, Ana García-Campos, Octavio Cordova-Arevalo, Margarita Mendoza-Pintos, Sergio Cinza-Sanjurjo, Manuel Portela-Romero, Pilar Mazón-Ramos, Jose Ramon Gonzalez-Juanatey

**Affiliations:** 1 Servicio de Cardiología, Complejo Hospitalario Universitario de Santiago de Compostela, Choupana s/n, 15706 Santiago de Compostela, Spainpilarmazon@yahoo.es (P.M.-R.);; 2 Instituto de Investigación Sanitaria de Santiago de Compostela (IDIS), Choupana s/n, 15706 Santiago de Compostela, Spain; 3 Centro de Investigación Biomédica en Red-Enfermedades Cardiovasculares (CIBERCV), Av. Monforte de Lemos, 3-5. Pabellón 11. Planta 0, 28029 Madrid, Spain; 4 Department of Business, University of Vigo, 36310 Vigo, Spain; octavio.oficina@gmail.com; 5 Department of Pharmacology, Pharmacy and Pharmaceutical Technology, R+D Pharma Group (GI-1645), Faculty of Pharmacy, Health Research Institute of Santiago de Compostela (IDIS), University of Santiago de Compostela, 15782 Santiago de Compostela, Spain; 6 Departamento de Economía Financiera y Contabilidad, Facultad de Ciencias Económicas y Empresariales, Universidad de Santiago de Compostela, Av. Burgo, s/n, 15782 Santiago Compostela, Spain; 7 Medtronic España, S.A., 15753 Santiago de Compostela, Spain; 8 CS Milladoiro, Área Sanitaria Integrada Santiago de Compostela, 15895 Travesía do Porto, Spain; 9 CS Concepción Arenal, Área Sanitaria Integrada Santiago de Compostela, Rúa de Santiago León de Caracas, 12, 15701 Santiago de Compostela, Spain

**Keywords:** computed tomography, magnetic resonance imaging, cardiology, cost measures, cost analysis

## Abstract

Introduction: In recent years, several hospitals have incorporated MRI equipment managed directly by their cardiology departments. The aim of our work is to determine the total cost per test of both CT and MRI in the setting of a Cardiology Department of a tertiary hospital. Materials and Methods: The process followed for estimating the costs of CT and MRI tests consists of three phases: (1) Identification of the phases of the testing process; (2) Identification of the resources consumed in carrying out the tests; (3) Quantification and assessment of inputs. Results: MRI involves higher personnel (EUR 66.03 vs. EUR 49.17) and equipment (EUR 89.98 vs. EUR 33.73) costs, while CT consumes higher expenditures in consumables (EUR 93.28 vs. EUR 22.95) and overheads (EUR 1.64 vs. EUR 1.55). The total cost of performing each test is higher in MRI (EUR 180.60 vs. EUR 177.73). Conclusions: We can conclude that the unit cost of each CT and MRI performed in that unit are EUR 177.73 and EUR 180.60, respectively, attributable to consumables in the case of CT and to amortization of equipment and staff time in the case of MRI.

## 1. Introduction

The constant increase in healthcare spending by the most developed countries, due to various factors such as the ageing of the population or the high cost of technological advances in this field, has raised doubts about the sustainability of public healthcare systems [1,2]. Economic evaluation in healthcare has thus become essential when it comes to allocating limited healthcare resources to achieve the maximum possible benefit, in this case people’s health, at the lowest cost, helping in the design and choice of healthcare policies that specify the healthcare needs to be covered, the degree of coverage provided and the type and volume of resources to be used [3].

From the clinical point of view, in recent decades, the use of waveform imaging has become more widespread. One of the fields where its use is growing the most is cardiology, especially diagnosis by computed tomography (CT) and magnetic resonance imaging (MRI), mainly because they are non-invasive tests that provide versatility and the capacity to produce detailed images of the heart, which favors precision in diagnosis; they are also currently the reference tests for the study of the morphology and functioning of the heart [4,5].

This widespread use and the increase in tests requested make more efficient use of cardiac imaging mandatory in a multidisciplinary unit in which cardiologists and radiologists participate and that work exclusively for the diagnosis of cardiovascular pathology. In this search for efficiency, the Spanish Society of Cardiology and the Spanish Society of Medical Radiology published a joint document in 2016 in which they proposed the creation of multidisciplinary cardiac pathology committees in charge of establishing protocols to improve the quality and efficiency of the magnetic resonance [6].

CT and MRI have different advantages and disadvantages, and therefore the indication to perform one exam or the other depends on what is needed to be evaluated and what cardiac pathology is being looked for or suspected. Thus, computed tomography, due to its high spatial resolution, allows adequate visualization of structures as fine as the coronary arteries, and can also evaluate cardiac function and the rest of the cardiac and thoracic anatomy. MRI has high temporal resolution, making it a great test for assessing cardiac function. It provides information on perfusion, myocardial viability, areas of fibrosis, abnormal deposition of substances in the myocardium. It also performs flow measurements, with which it is possible to quantify valvular pathology and evaluate functional compromise in operated congenital pathologies [7,8]. It must be said that for some authors, CT and MRI technologies are complementary rather than substitutive [9].

Faced with the pressing need to reduce waiting times and increase the number and quality of tests, several hospitals have in recent years incorporated MRI equipment managed directly by their cardiology services. In this regard, a study by Barreiro Pérez et al. reports data from the first CMR unit installed in Spain which began operating in July 2014 at the University Hospital of Salamanca. This work yields a series of results similar to those of other studies carried out in Germany and the United Kingdom, the main conclusion being that the installation and management of an MRI unit in the cardiology department of a hospital improves care practices and helps to reduce waiting lists. In addition, it enables the effective participation of the service’s professionals, improves training and favors research [5].

In 2019, the Hospital Clínico Universitario located in the Euroregion Galicia–North Portugal implemented an imaging unit with MRI and CT equipment managed directly by the cardiology service; it is called the Advanced Cardiovascular Imaging Unit (UICVA). The main objective of this manuscript is to determine the total cost per test of both CT and MRI in the setting of a Cardiology Department of a tertiary hospital.

## 2. Materials and Methods

The process followed for estimating the costs of CT and MRI tests consists of three steps:

1. Identification of the stages of the testing process: after analyzing the general process through which testing is carried out, the following stages were identified:
  (a) Requesting the test: regardless of the patient’s origin (outpatient, inpatient or unscheduled), the process begins with a request by a cardiologist or surgeon for an MRI or a CT scan.
  (b) Assessment and planning of requests: requests are assessed and filtered to determine the appropriateness of the test by a cardiologist with expertise in advanced cardiovascular imaging. In this way, a better selection and prioritization of requests is achieved, avoiding the unnecessary performance of complementary tests that do not involve changes in clinical decision-making [10].
  (c) Appointment and scheduling of patients: an administrative staff member makes appointments for outpatients.
  (d) Conducting the test: this stage is further subdivided into several parts:
    i. Preparation of the patient. The patient is received in the UICVA by the nursing staff. Patient preparation includes weighing and measuring the patient, arranging the signing the informed consent form, administering the necessary premedication and positioning the patient on the stretcher.
    ii. Imaging: performed by radiology technicians and nurses.
    iii. Removal of the patient by the nurse and the radiology technician.
    iv. Observation time, for patients who have had contrast administered, for 30 min.
  (e) Preparation of the report by a cardiologist and a radiologist.
2. Identification of the resources consumed in the performance of the tests: cost analysis was carried out from the perspective of the health sector [11] so that the resources, material and human, necessary to carry out the tests [12] are taken into account, without including the costs supported by the patient (travel to the hospital, loss of working hours, etc.) or social costs, which usually only add complexity without modifying the results already provided by the previous analysis [13]. The costs incurred in the different phases of the testing process are classified by distinguishing between personnel costs (by professional categories: medical, non-medical healthcare and non-healthcare) and operating costs (consumables, equipment and others). In turn, within each category, a subdivision is made according to the nature of the resources consumed.
3. Quantification and valuation of consumption: Once the different types of resources consumed in carrying out the tests are identified, they must be measured (in physical units) and valued (in monetary units).
  (a) Personnel costs: in this case, it is necessary to establish the average time spent by each professional involved in the performance of the tests [14]. To this end, the time spent by the different workers involved in each of the phases related to each test was timed for a random sample of 36 patients attending the Hospital Clínico Universitario de Santiago in April and May 2021. Given that both MRI and CT are used in different protocols, we collected the data corresponding to each of them; although, as there were no significant differences, we opted to estimate the overall average time. In addition, given the impossibility of timing some of the phases, the times were standardized according to the data published by the National Institute of Health (INSALUD). Table 1 summarizes the average times obtained for each phase of each test.
    In regard to the times showed in Table 1, it should be noticed that given the configuration of the UICVA and the established protocols, the X-ray technician has to control the way in which the process is carried out, and it is not possible to acquire several images at the same time or perform another specific task, so this time includes an opportunity cost. However, it should be noted that our times (and therefore our personnel costs) are significantly lower than those obtained in other studies, such as that of Ten Roldán et al., which makes us assume that it is a common practice in the Spanish healthcare system [15]. On the other hand, the consultants only make the request for the test, while the coordinating cardiologist assesses the appropriateness of the request and makes the final report.
    Once the time spent by the different professionals in the performance of each test is measured, we proceed to their valuation for which, following Alonso Alperi, we base ourselves on the gross salaries corresponding to each professional category and the annual working day [14]. Based on the data provided by the Euroregion Galicia–North Portugal for the year 2022, the cost per hour for each professional category is calculated. The calculation of the costs of the personnel involved in each of the phases/activities associated with the performance of the tests is carried out by standardizing the cost by 1 h time periods.
  (b) Cost of fungibles: with regard to the evaluation of the consumption of fungible materials (medical material, non-medical material and pharmaceutical products), based on the data provided by the hospital itself, we determined a total cost of fungibles of EUR 129,382.28 in the case of CT and EUR 31,004 for MRI. The main difference between the two is due to pharmacological products (EUR 113,610.28 vs. EUR 15,232).
  (c) Equipment costs: three components are distinguished within these costs: depreciation cost, operation and maintenance cost and opportunity cost.
    i. For the calculation of the depreciation cost, the use of a financial method of depreciation is recommended in healthcare economic evaluation [13,16]. Furthermore, an estimated useful life of 8 years is considered for radiological equipment as this is the useful life generally assigned to this equipment [17] with a residual value of zero. As for the interest rate, we chose a discount rate of 3% as this is the rate commonly used in the literature [14,16].
    ii. With regard to the cost of operation and maintenance of the equipment, in the case of our hospital, this cost usually ranges between 7% and 8% of the value of the investment, so for the present analysis, we use the average of both (7.5%) [14].
    iii. We refer to the opportunity cost of the capital invested in this equipment. Its consideration seeks to reflect the opportunity loss of investing that capital in other projects by applying an interest rate to the total capital invested. In the specific case of the Galicia–Northern Portugal Euroregion, there is no such opportunity cost since the technological park installed in the northern geographical area of the Galicia–Northern Portugal Euroregion is one of the oldest in Spain and therefore any renewal or incorporation is necessary [18].
    iv. The cost of fixed assets (i.e., equipment) is calculated taking into account which part corresponds exclusively to the Cardiology Service and the proportion that corresponds to each test: 42% of the tests performed per year correspond to MRI and the remaining 58% correspond to CT. We distinguish between specific equipment for each type of test and general equipment. In regard to the former, it includes Revolution CT GEHC Computed Tomography and CT contrast equipment for CT and MRI “Signa Architect 3.0 T” and MRI contrast equipment for MRI. General equipment includes arrest trolley, defibrillator, infusion pumps, sphygmomanometer, pulse oximeter, IV/multifunction trolleys, oxygen flowmeters, suction vacuum gauges, patient chairs, patient chairs, computers, etc. Both categories are considered in the calculation of the annual depreciation cost.
      Firstly, we compute the annual depreciation cost by taking into account the purchase price of the equipment, an estimated life of 8 years, and a discount rate of 3%. Since the equipment is not used exclusively by the cardiology service, it is necessary to determine the portion of the cost of amortization that corresponds to the tests under study. To make this allocation, we consider that the most appropriate allocation key is the time of use of the equipment. Therefore, we divide the annual depreciation cost over the annual hours of use of the equipment and then multiply the cost per hour by the number of hours that the cardiology service used the equipment. In addition, the annual depreciation cost of the remaining equipment corresponding to the cardiology service is divided between CT and MRI depending on the number of tests performed (i.e., in this case, we make a second distribution using the percentage represented by each type of test with respect to the total number of tests corresponding to the cardiology service as the distribution key). Finally, the annual depreciation cost obtained for CT and MRI is divided by the total number of tests performed to obtain the cost per test.
  (d) Overhead costs: these are estimated by distributing the overhead costs (electricity, water, etc.) in our hospital, allocating the corresponding amount to the imaging tests according to the physical space within the hospital and the number of tests performed.
  (e) Social costs:
    (1) The cost of the trip (which corresponds to the patient’s displacement to the hospital) is calculated from the average distance between the parish to the hospital. In the territory of GOVERNMENT DECRET, the average is set at 3.92 km [19], which is multiplied by 0.19 euros/km to calculate the cost of this distance (Presentation for the Workshop on Regional Funding, 2015).
    (2) The cost of the time invested in the trip to the hospital to attend the exploration is estimated from the weighted mean time for our health area (39,084 min). To this time, the average duration of the exploration activities is added (157.23 min for CT and 188.58 min for MRI), as well as the time to park and arrive at the consultation area (another 20 min), so the total time is 177.23 min for CT per patient and 208.58 min per patient for MRI.
      - For the working-age population, the cost is estimated taking into account the average gross income of the worker (18,768.21 euros/year) and the unemployment rate (12%) in our health area as of 31 December 2022) [20]. On the other hand, leisure time is valued at 47% of the cost of working hours [21].
      - For the retired population, the cost is estimated taking into account the percentage of people aged 65 or over who perform volunteer work according to the CIS-IMSERSO study (2.3%) [22]; those dedicated to caring for grandchildren according to the study “Living Conditions of the Elderly” carried out by the Sociological Research Center (22.6%) [23], and the Public Indicator of Multiple Effects Income (IPREM).
    (3) The cost of time on the waiting list is analyzed based on the study by Propper et al. [24], valued at GBP 38.89 (in 1987) for each month.
      Transferred to 2022 in euros, they correspond to EUR 128.30.
    (4) Waiting time (as of 31 December 2022) for CT scanning in the Radiology Department: 79.8 days; the Cardiology Department: 24.4 days. For MRI, the waiting time in the Radiology Department: 65.2 days; the Cardiology Department: 24.4 days.
    (5) The cost of pollution from private motor vehicle travel by road. A significant part of transport studies focuses on carbon dioxide (CO_2_) emissions and, in turn, CO_2_ emissions are directly related to fuel consumption and fuel efficiency [20,21]. Chester and Horvath (2008) estimate that the total emissions of a passenger car are 0.36 kg CO_2_ per passenger and mile [25]. The cost of CO_2_ emissions is EUR 0.0770235 per gram [26].

The Cobb–Douglas function presents the following form [27]:

$$TC=\alpha X_{1}^{\beta_{1}}X_{2}^{\beta_{2}}\cdots X_{n}^{\beta_{n}},$$

where *X*_1_, *X*_2_ and *X_n_* represent the total cost and the different costs, and *α*, *β*_1_, *β*_2_ and *β_n_* are constants.

The model was linearized by calculating the natural logarithms on both sides of the equality:

$$\ln TC=\beta_{0}^{}+\beta_{1}^{}\ln X_{2}^{}+\beta_{n}^{}\ln X_{n}^{}$$

For the two models ((1) CT Costs; (2) MRI Costs), the equations proposed are the following:

ln *TCCT* = *β*_0_ + *β*_1_ ln *Cost of Staff* + *β*_2_ ln *Cost of fungible materials* + *β*_3_ ln *Cost of equipment*, (1)

ln *TCMRI* = *β*_0_ + *β*_1_ ln *Cost of Staff* + *β*_2_ ln *Cost of fungible materials* + *β*_3_ ln *Cost of equipment.* (2)

Each of the Beta coefficients of the previous function represents the partial elasticities of each variable, showing the weight of each variable in the total cost. It allows evaluating the possible existence of economies of scale (*EE*). The economies of scale are calculated by adding the partial derivatives of the cost function:

*EE < 1 presence of economies of scale*,

*EE = 1 constant returns to scale*,

*EE > 1 presence of diseconomies of scale*.

In order to overcome the risk of overfitting due to the small sample obtained (although it represents 100% of the universe of patients in the hospital under study), the Cobb–Douglas regression was performed with a bootstrapping procedure. Bootstrapping is an internal validation method that is ideal for small samples [28]. The aim of bootstrapping is to replicate this procedure by sampling within the study population, with replacement to create more learning subgroups [29]. In each bootstrap sample, data are analyzed as in the original study sample, repeating each step of model development, including predictor selection strategies. The bootstrap sample repetition process was performed 10,000 times [30].

## 3. Results

A total of 565 MRI and 1018 CT scans were performed in 2021. As indicated above, the costing process started by identifying the different types of resources consumed in performing the tests (e.g., staff, consumables, equipment, general costs); then, consumption was quantified and valued in monetary units by applying different valuation criteria. The results are presented in Table 2, which shows the costs for both tests in the Cardiology Department.

For each test, consumables represent the highest expenditure in CT (52.49% of the total) due to the high unit cost of the contrast used for the tests (accounting for more than 92% of the total in this category). In the case of MRI, the greatest expenditure is generated by equipment (49.82%), fundamental amortization of equipment (46.74%), and staff time (36.56%), which is mainly the time of medical staff (22.02%) and non-medical staff (14.27%) due to the longer duration of the test.

In Table 2, the personnel involved in the tests was classified according to the criteria established by the hospital, differentiating three categories: medical staff (consultant and coordinating cardiologist), non-medical staff (nurse and X-ray technique) and other (secretary). The low cost of “Other” is due to the fact that the secretary’s salary is low and they spend little time on the tests under study.

The cost structure of both tests was compared as the total cost is similar (approximately EUR 180/test), although the composition of the cost structure is very different. MRI involves higher personnel (EUR 66.03 vs. EUR 49.17) and equipment (EUR 89.98 vs. EUR 33.73) costs, while CT requires higher expenses in consumables (EUR 93.28 vs. EUR 22.95) and overheads (EUR 1.64 vs. EUR 1.55). The total cost of each test is higher for MRI (EUR 180.60 vs. EUR 177.73). As can be seen, the amortization of the equipment is more important in MRI (46.74% vs. 17.80%) due to its higher cost and time of use throughout the working day. In CT, it is drugs that represent the greatest expense for each test (48.57% vs. 8.86%). On the other hand, in both tests, general costs, the cost of non-medical consumables and the cost of non-medical personnel have a very small influence on the total cost of the tests.

Table 3 shows the results of the Cobb–Douglas function for the two models. In the first model (CT), the variables with the highest level of explanation of the total cost are Cost of Equipment and Cost of Staff. With respect to the second model (MRI), the variable that explains the total cost to a greater extent is the Cost of Staff associated with the Cost of Equipment. It should also be noted that the cost of consumables has a greater weight in the MRI cost function than in TC. Because of the singular contribution of MRI to functional studies, we can hypothesize that the greater impact of specialized personnel costs in the MRI function compared to that of CT may be due to the dual ability of MRI to perform both morphological and functional studies.

Due to the unique contribution of MRI to functional studies, we can hypothesize that the greater impact of specialized personnel costs in the MRI function compared to CT may be due to the dual ability of MRI to perform both morphological and functional studies. In the case of the costs associated with the consumption of consumables, the difference in the influence on the final cost is mainly due to the contrast used, iodine for CT and gadolinium for MRI.

Based on the results of the Durbin–Watson test, there are no autocorrelation problems for any of the two models. Likewise, the hypothesis of non-existence of collinearity is accepted.

Regarding the existence or not of economies of scale (*EE*), diseconomies of scale are observed both in Model (1) for CT and Model (2) for MRI. 

The fact that r is greater than one suggests that despite the size of these units and the conditions under which they operate, the possibility of lowering their production costs is not absent in the production process because they operate at sub-optimal points. This means that if production can be raised, they can approach the minimum efficient scale of the equipment.

Table 4 shows the societal costs per patient for each scan, both CT and MRI. The costs are the same except for the cost of the total scan time. It is striking that the highest amount of these costs is the contamination costs for travel to the hospital for the tests.

Finally, a quantifiable benefit is the reduction in waiting times for examinations if CT and MRI scans are managed by the Cardiology Department compared to the Radiology Department. Thus, the cost of the waiting time in Radiology for CT would be EUR 341.27, and for MRI it would be EUR 278.83, while the waiting time in the Cardiology Service for both technologies would be EUR 104.35.

## 4. Discussion

The results of our study analyze the costs associated with the performance of CT and MRI by our Cardiology Department at the University Hospital located in the Galicia–North Portugal Euroregion, analyzing the flow of patients and quantifying the consumption of resources that provided the final calculation of the cost of each test. Our results confirm that both tests have a very similar cost, around EUR 180, but this cost, while in CT is mainly due to the use of contrasts, in MRI it is due to the amortization of the devices, which is much more expensive, and staff time due to the longer duration of the test.

Although the total cost/test ratio is similar, there are important differences in the composition of the direct costs of performing the two tests which have relevant implications for the economic and medical management of the service. For example, we see that the cost of equipment is much higher for MRI than for CT (its weight in the total cost is almost three times higher for MRI compared to that for CT) due to the higher cost of MRI equipment, but also to the fact that this equipment is used by the cardiology service for more hours per day than CT equipment. In addition, MRI has higher personnel costs than CT. As these are both fixed costs and therefore difficult to change in the short term by healthcare managers, these differences should be known and analyzed from the point of view of service management in order to improve operability (less waiting time, less repetition of tests, better triage, etc.). On the other hand, the fact that in the case of CT the main cost component is the consumption of consumables (a variable cost on which health managers have a greater capacity to act) allows a very different approach to be taken when making decisions on the management of the service. Ultimately, it is the analysis of the composition of the costs of each test, rather than the overall result, that allows managers to improve their decision-making in this area.

After a thorough literature review, we found that our results represent a better approximation to the costing of both tests, as discussed below, and therefore a valuable source of information for healthcare managers. In any case, it should be borne in mind that the results of the economic evaluation do not constitute a decision rule, but should only serve as a guide to decision-making, and it is very important to decide the clinical role that each complementary test plays in the decision-making process of the specialist attending the patient. In the particular case of coronary artery disease, the main indications for which CT and MRI are used are various diagnostic strategies such as coronary angiography, which are available in addition to CT and MRI. In clinical practice, 60% of angiographies do not show evidence of coronary lesions, and therefore entail a cost and, above all, a risk for the patient as it is an invasive test, so it is very important to identify the correct sequence of complementary tests based on their efficacy, safety and cost [31].

Our results represent a better approximation than conventional cost analyses that are based on theoretical models and that globally assume the costs attributed to a given syndrome, such as the work of Yin Ge in patients with chronic ischaemic heart disease [32], without taking into account the explicit costs of each test performed individually. In this sense, our work adds the value of obtaining all the costs explicitly associated with the patient flow in the diagnostic process of both CT and MRI managed by a Cardiology Service, providing real results and not those based on cost assumptions. In this sense, Terpenning et al. are critical of analyses such as ours, as they consider that real costs may be dependent on external factors, such as practice, location of the center, inpatient or outpatient setting or the role of insurance companies in countries such as the United States [33] 17; however, although we recognize that our analysis could probably be replicated in each country, and we believe that the actual costs may be dependent on external factors, such as practice, location of the center, inpatient or outpatient setting or the role of insurance companies in countries such as the United States [33], our analysis should probably be replicated in each individual health system to adapt it to its real environment. Such approach is much closer to reality, and decision-making including the efficiency of diagnostic systems will be more correct based on a model such as ours and not on a more theoretical model in which theoretical average costs are imputed, as in the work of Kozor et al. [34].

This type of economic analysis directs expenditure towards elements that do not generate it, at least not in such a significant way. Our study showed that CT scans cost EUR 177.73 each, mainly attributable to the consumption of consumables (which accounted for 54.49% of the total), followed by the cost of personnel (27.67%). However, other studies such as that of Ten Roldán et al., carried out in Valencia and based on theorized models, showed that most of the cost was generated by personnel, constituting 72% of the total cost, followed by equipment (16% of the total), while consumables only represented 9% of the cost of the test; this difference cannot be justified simply by the difference in remuneration as it is not justified between two Health Services in our country, so the difference lies in the different economic model used for the analysis. On the other hand, in a similar analysis, Yarahmadi et al. concluded that most of the cost corresponds to the depreciation of capital equipment attributable to equipment breakdowns, disproportionate and excessive use of equipment and equipment maintenance [35], which also does not seem to be in line with reality, especially in a health service such as the Galician one, where the equipment is the oldest in Spain [18].

In the case of MRI, the unit cost was EUR 180.60, very similar to that of CT, but this cost was generated by equipment depreciation (49.82%) and staff time (36.56%). Here, again, we find discrepancies due to the use of simulation-based analytical accounting models such as Centone et al. who, despite including direct costs and overhead costs, without taking into account indirect costs, obtained a higher unit cost per test than ours [36]. Our argument that simulation models are overused without recourse to real data is reinforced by a recently published systematic review by Pandya et al., which concludes that the majority of published studies are actually based on simulation models [37].

In a study by Paquette and Lin [38], the total reduction in emissions of environmental pollutants from passenger cars, including carbon dioxide, carbon monoxide, nitric oxides and volatile organic compounds, was 1632 kg, 42.867 g, 3160 g and 4715 g, respectively, with a total of 194 gallons of gasoline saved; although, unlike our study, it does not incorporate an economic quantification of the cost or savings of reducing the level of air pollution.

In view of the above, we consider that our cost analysis is methodologically much more solid than what has been published to date, including indirect costs and general costs which not all authors agree to include [11] 8 but which undoubtedly enrich and give solidity to the conclusions obtained [15]. Moreover, the interest in this type of analysis is growing, as healthcare spending has increased in recent decades in developed countries, making efficient resource management a priority. In this sense, given the lack of similar studies that perform an economic analysis of CT and MRI in cardiology due to the novelty of a cardiology service directly managing an imaging unit, the present study is a first approximation to the calculation of the costs of these tests and, therefore, a valuable source of information for health managers.

Despite these strengths, our work is not without limitations. On the one hand, sometimes, aspects such as the management of test requests, the assessment of test indications, or the preparation of reports had to be estimated on the basis of the data published by INSALUD, as it was impossible to measure them; we do not believe that this limitation invalidates our results, as we used the data published by the Health Service itself; in any case, we could be somewhat more precise, but we do not consider that this would significantly change the results.

Undoubtedly, our work opens the door to future analyses, such as the comparison of costs in the current UICVA compared to those generated in the performance of the same tests before the creation of the Unit, even if its creation was not aimed at reducing these costs. Another element to be added would be the analysis of the costs borne by the patient, as well as the social costs, which in this study we have decided to eliminate as mentioned above. Finally, another possible extension of the present analysis should include a cost-benefit or cost-effectiveness analysis, taking into account the medical and economic outcomes (effects) that improve the patient’s care process [31].

## 5. Conclusions

In light of the above, we can conclude that in the context of the growing importance of economic evaluation in the healthcare field for decision-making, our work represents a first approximation to the calculation of costs associated with the performance of CT and MRI scans managed by a Cardiology Department and a valuable source of information for healthcare managers. Furthermore, we were able to conclude that the unit cost of each CT and MRI scan performed in this unit is EUR 177.73 and EUR 180.60, respectively, attributable to consumables in the case of CT scans and to the amortization of equipment and staff time in the case of MRI scans.

## Figures and Tables

**Table 1:** Average times taken by staff to carry out the tests.

Stage	Staff	Time Spent	
		CT	MRI
Request for test	Consultant	45′	45′
Assessment of the test	Coordinating cardiologist	30′	30′
Patient appointment	Secretary	2′32″	2′32″
Preparation of the patients	Nurse	12′57″	17′24″
	X-ray technician	9′31″	7′51″
Image acquisition	Nurse	46″	-
	X-ray technician	7′45″	1 h 21′43″
Removal of the patient	Nurse	2′29″	2′54″
	X-ray technician	2′29″	2′54″

h: hours, “′”: minutes; “″”: seconds.

**Table 2:** Cost calculation per CT and MRI test.

CONCEPTS	CT		MRI	
Staff	EUR 49.17	27.67% (100%)	EUR 66.03	36.56% (100%)
Consultant	EUR 39.77	22.38% (80.88%)	EUR 39.77	22.02% (60.23%)
Nursing	EUR 8.91	5.01% (18.12%)	EUR 25.77	14.27% (39.02%)
Other	EUR 0.49	0.28% (1.00%)	EUR 0.49	0.27% (0.75%)
**Consumables**	EUR **93.28**	**52.49% (100%)**	EUR **22.95**	**12.71% (100%)**
Medical devices	EUR 6.95	3.91% (7.45%)	EUR 6.95	3.85% (30.27%)
Non-Medical devices	EUR 0.01	0.003% (0.006%)	EUR 0.01	0.003% (0.023%)
Pharmacy	EUR 86.33	48.57% (92.55%)	EUR 16.00	8.86% (69.70%)
**Equipment**	EUR **33.73**	**18.98% (100%)**	EUR **89.98**	**49.82% (100%)**
Depreciation	EUR 31.64	17.80% (93.83%)	EUR 84.42	46.74% (93.83%)
Maintenance	EUR 2.08	1.17% (6.17%)	EUR 5.56	3.08% (6.17%)
**General Costs**	EUR **1.55**	**0.87%**	EUR **1.64**	**0.91%**
**Cost per test**	EUR **177.73**	**100.00%**	EUR **180.60**	**100.00%**

CT: Computerized Tomography; MRI: magnetic resonance imaging. In bold is represented the total of the following items.

**Table 3:** Co–b-Douglas cost function analysis type of patient.

	CT	MRI
Constant	−12.299 (0.002)	−38.376 (0.008)
Cost of Staff	31.100 (0.000)	35.826 (0.000)
Cost of fungible materials	21.756 (0.000)	25.214 (0.000)
Cost of Equipment	38.662 (0.000)	31.296 (0.000)
F	0.000	0.000
R^2^	0.883	0.970
DW	1.711	1.636

**Table 4:** Social costs per test.

Social Costs	CT	MRI
Travel	8.99	8.99
Test Time	14.13	16.61
Waiting Time	104.35	104.35
Pollution	879.08	879.08

## Data Availability

No new data were created.
